# Possible Influences of Endogenous and Exogenous Ligands on the Evolution of Human Siglecs

**DOI:** 10.3389/fimmu.2018.02885

**Published:** 2018-12-04

**Authors:** Takashi Angata

**Affiliations:** Institute of Biological Chemistry, Academia Sinica, Taipei, Taiwan

**Keywords:** Siglec, sialic acid, Neu5Ac, Neu5Gc, immunity, microbes

## Abstract

Sialic acids, a group of acidic sugars abundantly expressed in the tissues of deuterostome animals but rarely found in microbes, serve as a “signature of self” for these animals. Cognate sensors for sialic acids include Siglecs, a family of transmembrane lectins of vertebrate immune systems that recognize glycans containing sialic acids. A type of sialic acid called *N*-glycolylneuraminic acid (Neu5Gc) is abundant in many mammalian lineages including great apes, the closest extant relatives of modern human, but was lost in the lineage leading to modern human via the pseudogenization of the *CMAH* gene encoding the enzyme that converts *N*-acetylneuraminic acid (Neu5Ac) to Neu5Gc. Loss of Neu5Gc appears to have influenced the evolution of human Siglecs, such as the adjustment of sialic acid binding preferences and the inactivation of at least one Siglec. In addition, various mechanistic studies using model systems and genetic association studies have revealed that some human Siglecs interact with pathogens and influence the outcome of infections, and these pathogens in turn likely influence the evolution of these Siglecs. By understanding the evolutionary forces affecting Siglecs, we shall achieve a better appreciation of Siglec functions, and by understanding Siglec functions, we can obtain deeper insight into the evolutionary processes driving Siglec evolution.

## Introduction

The role of immunity is to distinguish self vs. non-self (or what is not dangerous vs. dangerous) and to eliminate or contain the latter. Various biomolecules (nucleotides, peptides, lipids, polysaccharides, and their combinations) can be a signature of non-self (i.e., pathogen-associated molecular patterns; PAMPs), as exemplified by the diversity of ligands for Toll-like receptors, C-type lectin-like receptors, RIG-I-like receptors, and NOD-like receptors, all of which work as “pattern-recognition receptors” (1–4). Meanwhile, the signature of self (i.e., self-associated molecular patterns; SAMPs) is less well-understood, but some glycoconjugates would qualify as such (5, 6). Sialic acids are commonly synthesized by deuterostome animals and displayed on the cell surface in abundance but are rare in microbes (7), making them an ideal SAMP for distinguishing self- vs. non-self (5, 6).

**Figure 1:**
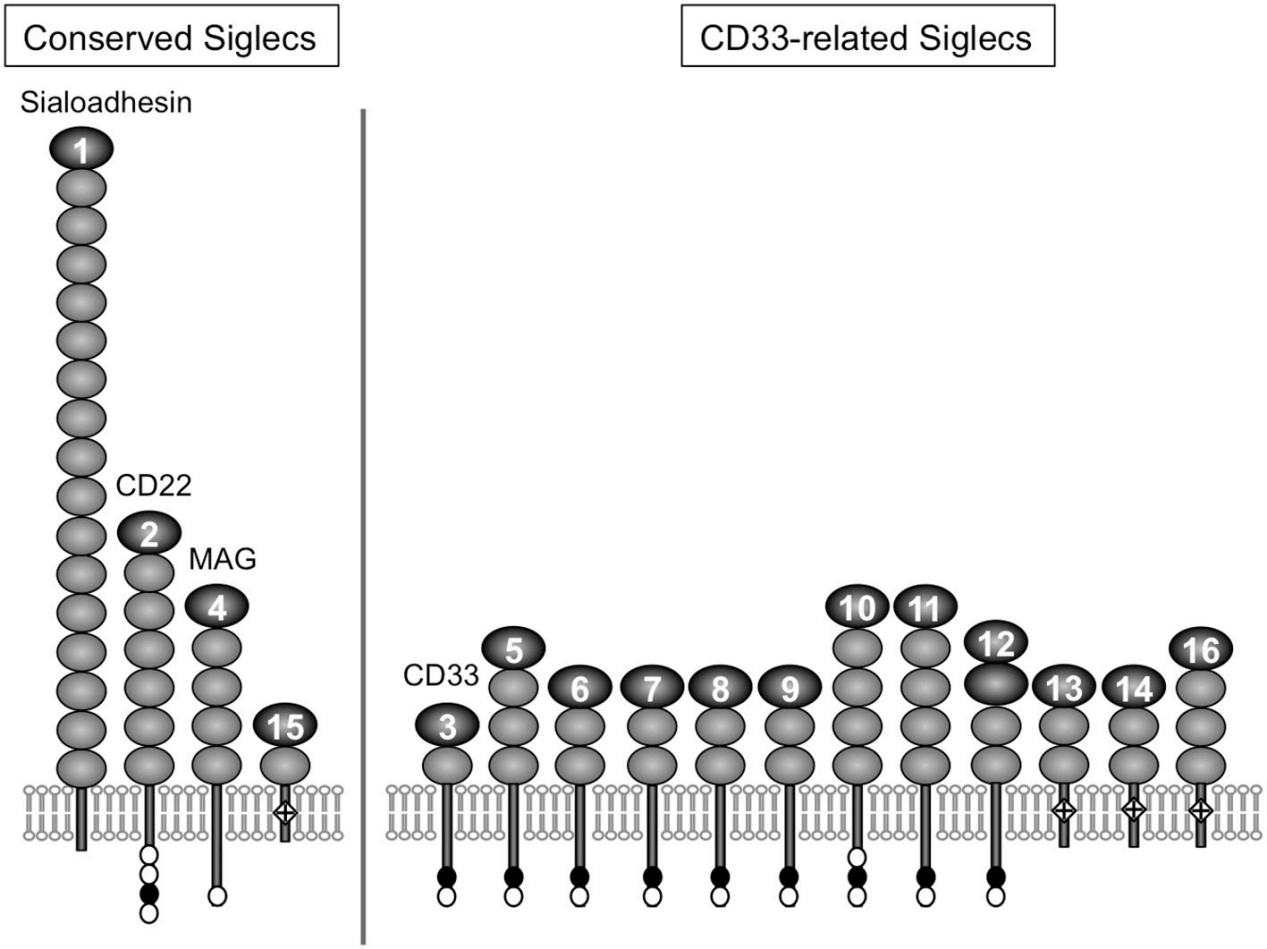
Illustrative representation of ape Siglecs. Mammalian Siglecs can be classified as relatively conserved members (Sialoadhesin/Siglec-1, CD22/Siglec-2, MAG/Siglec-4, and Siglec-15) and less conserved members (CD33-related Siglecs). CD33-related Siglecs are encoded in the gene cluster (15). Siglecs can be also classified based on the partner molecule involved in downstream signal transduction (i.e., those that have ITIM and interact with tyrosine phosphatase SHP-1, and those that have positively charged amino acid residue in the transmembrane domain and interact with adapter molecule DAP12, which has the “immunoreceptor tyrosine-based activating motif“ (ITAM) and recruits tyrosine kinase Syk). Siglec-13 is missing in humans but present in apes and old-world monkeys (15). Modified from (16). Closed circle, open circle, and diamond with + mark in the figure represent ITIM, ITIM-like motif, and positively charged amino acid residue in the transmembrane domain (that is required for the interaction with DAP12), respectively.

For a chemical entity to be a molecular signature of self or non-self for the immune system, there must be a sensor that recognizes it. For sialic acids, Siglecs appear to be the primary pattern-recognition receptors (8–11). Siglec is a composite word from “sialic acid,” “immunoglobulin (Ig) superfamily,” and “lectins” (12). The Siglec family appears to be present only in vertebrates (13, 14). Siglecs are type 1 transmembrane proteins, with an extracellular domain consisting of multiple Ig-like domains (of which the N-terminal Ig-like domain is primarily responsible for the recognition of sialoglycans), followed by a single-pass transmembrane domain and cytoplasmic tail (Figure 1). Most of the known mammalian Siglecs are expressed on leukocytes and have an intracellular sequence motif called the immunoreceptor tyrosine-based inhibitory motif (ITIM) that recruits tyrosine phosphatase SHP-1 and thus transduces inhibitory signals. Thus, they are considered to function as sensors for sialic acids as a molecular signature of self. [However, there are some examples that imply this generalization may be somewhat too simplistic (17, 18).]. Although rodents are essential model animals for mechanistic studies in immunology, differences in primate and rodent CD33-related Siglecs (15) impose a significant challenge in the extrapolation of findings in rodents to human immunology. This situation parallels that of other immunoglobulin-like receptor families, leukocyte immunoglobulin-like receptors (LILR) and killer cell immunoglobulin-like receptors (KIR), that are encoded in a gene cluster on the same human chromosomal region as CD33-related Siglecs (chromosome 19q13.4) and are involved in self-recognition through interaction with MHC class I (19–21).

“Sialic acids” is a collective term for various naturally occurring acidic sugars with a common nine-carbon backbone (22). *N*-acetylneuraminic acid (Neu5Ac) is the most common type of sialic acid, and its C5-hydroxylated derivative *N*-glycolylneuraminic acid (Neu5Gc), along with the derivatives of Neu5Ac and Neu5Gc (mostly modified at C4 and/or C7-C9 hydroxyl groups), are generally present in mammalian tissues (22). Neu5Gc is abundant in many mammalian species, whereas humans have lost Neu5Gc, owing to the mutation (exon deletion) of the *CMAH* gene encoding CMP-Neu5Ac hydroxylase that is solely responsible for the *de novo* biosynthesis of Neu5Gc from Neu5Ac (23–26). Although some bacteria have developed ways to synthesize Neu5Ac, so far no study has demonstrated the presence of Neu5Gc on microbes (27) [A recent genomic survey (28) reported the presence of *CMAH*-like sequences in several microbial genomes, including those of several *Helicobacter* species that may express sialic acids. However, their enzymatic function has not yet been investigated]. Thus, Neu5Gc appears to be a quintessential signature of self, which is only present on deuterostome cells and missing on microbes. Indeed, some rodent Siglecs show a strong preference toward glycans containing Neu5Gc (29–32), whereas some others show a strong preference toward Neu5Ac (29, 33, 34). This imposes a conundrum: if one loses the best signature of self, the immune system may become more prone to attack its own cells (i.e., autoimmunity). How did the immune system of the human ancestor cope with the consequences of the dramatic change in the sialic acid landscape (i.e., shift of “sialome” from Neu5Gc to Neu5Ac) on the cell surface?

One possible consequence of Neu5Gc loss in human (and a possible response to the consequential autoimmune-prone state, in the evolutionary time scale) was a series of changes involving Neu5Gc-specific Siglecs, such as re-adjustment of binding specificity to Neu5Ac and “forced retirement,” as explained in the following section.

## Possible Influences of Neu5Gc Loss on Human Siglecs: Altered Binding Specificities

To understand the consequences of a species-specific event, it is natural to compare the phenotypes between the closest relatives that have undergone the event or have not. For human, the obvious choice is great apes including chimpanzee, which is the closest extant relative of modern human. Several earlier studies have shown that at least some great ape Siglecs preferentially recognize Neu5Gc (35–37). More recent data using the sialoglycan microarray also showed that primate CD33-related Siglecs generally tend to prefer Neu5Gc (38). Reported preferences of human and chimpanzee Siglecs toward Neu5Ac and Neu5Gc are summarized in Table 1. Thus, the loss of Neu5Gc likely meant attenuation of the interactions between Siglecs and self-associated ligands in the human ancestor.

**Table 1:** Binding preferences (Neu5Ac vs. Neu5Gc) and lineage-specific mutations in human and chimpanzee Siglecs.

**Siglec**	**Human preference**	**Chimpanzee preference**	Human-specific changes in Ig1 and Ig2^*^	Chimpanzee-specific changes in Ig1 and Ig2^*^	References^**^
Sialoadhesin/Siglec-1	**Ac** >> Gc	ND	1, 1	1, 0	(39)
CD22/Siglec-2	Ac ≈ Gc	Ac ≈ Gc	2, 0	2, 0	(35, 39)
CD33/Siglec-3	Ac < **Gc** (weak preference)	Ac < **Gc** (weak preference)	2.5, 2	5.5, 0	(35, 38, 39)
MAG/Siglec-4	ND	ND	0, 1	0, 0	[for the glycan preference of rodent MAG, see (33, 34, 40, 41)]
Siglec-5	Ac < **Gc** (weak preference)	X (Arg mut)	7.5, 2	5.5, 1	(35, 38)
Siglec-6	Ac < **Gc**	ND	0, 1	3, 0	(35)
Siglec-7	Ac ≈ Gc	Ac < **Gc**	1.5, 0	3.5, 0	(37)
Siglec-8	ND	ND	0.5, 2	1.5, 3	[for the glycan preference of human Siglec-8, see (42)]
Siglec-9	**Ac** > Gc (weak preference)	Ac < **Gc** (weak preference)	4, 0	3, 1	(37, 38)
Siglec-10	Ac < **Gc**	ND	0, 1	2, 1	(Consortium for Functional Glycomics data)
Siglec-11	Ac < **Gc**	Ac < **Gc**	2.5, 1	2.5, 0	(43, 44)
Siglec-12	X (Arg mut)	Ac < < **Gc**	2, 2	1, 3	(36)
Siglec-13	X (absent)	Ac ≈ Gc	(Cannot be determined)	(Cannot be determined)	(45)
Siglec-14	ND (likely Ac < **Gc**, from Siglec-5 data)	X (Arg mut)	6.5, 1	4.5, 1	(38)
Siglec-15	**Ac** > Gc	ND (**Ac** > Gc)	2, 1	0, 1	Unpublished
Siglec-16	**Ac** > Gc	Ac < **Gc**	1.5, 0	5.5, 3	(43, 44)

*ND, not determined; X, cannot be determined (either the protein is absent in the species or is present but does not recognize sialic acid owing to the mutation of essential Arg residue); >> or <<, strong preference; > or <, preference; ≈, no preference; Arg mut: mutation of arginine residue that is essential for sialic acid recognition. Sialic acid (Ac, Neu5Ac; Gc, Neu5Gc) preferentially recognized by each Siglec is highlighted with underline and bold typeface*.

* *The numbers of human- and chimpanzee-specific amino acid changes were deduced by aligning the amino acid sequences of Siglec orthologs from human, chimpanzee, and orangutan. In case the lineage specificity of the amino acid change cannot be unambiguously determined (i.e., when the amino acid at one position was different in all three species), ”0.5 difference“ was assigned to both human and chimpanzee. For Siglec-12 with two V-set domains, amino acid changes in the N-terminal V-set domain (Ig1) were counted as those in ”Ig1,“ and those in the first C2-set domain (Ig3) were counted as those in ”Ig2.“ Note that the ”species-specific changes“ were counted based on a reference sequence of human Siglecs and ”best hit” putative protein sequences in chimpanzee and orangutan by BLASTP search, without considering the polymorphisms in each species*.

** *The majority of the references in this table are reports that directly compare human and chimpanzee Siglec binding preferences. Note that different methods for analyzing Siglec–glycan interactions, such as glycan microarray vs. polymer-based probe binding, or even between different formats of glycan microarrays, may yield results that are not fully consistent in some cases*.

One Siglec that may have been substantially affected by the loss of Neu5Gc in the human ancestor is Siglec-12 (36). Chimpanzee Siglec-12 and human Siglec-XII are expressed on macrophages and lumenal epithelia (36, 46). Human Siglec-XII has a universal mutation (R122C) that makes the protein unable to recognize sialic acids (36). [Roman numerals are used for primate Siglecs that have a mutation at the essential arginine residue required for sialic acid recognition and thus cannot recognize sialic acid (15)]. Arginine-restored human Siglec-XII, as well as chimpanzee Siglec-12, strongly prefers Neu5Gc over Neu5Ac (36). In addition, some human *SIGLEC12* alleles have acquired additional mutations (stop codon, rs16982743, and frame-shift, rs66949844) that cause premature termination of Siglec-XII protein synthesis (36, 46). These “null” mutations are common in the modern human populations (global frequency of “null” alleles: 0.19 for rs16982743, 0.59 for rs66949844). These results imply a scenario in which a Siglec that lost an endogenous ligand was forced to “retire” and then is further getting eliminated. Given that the R122C mutation is fixed in modern human populations, it is tempting to speculate that the presence of functional “Neu5Gc-recognizing” Siglec-12 may have caused a disadvantage in ancestral humans. For example, zoonotic infection of some Neu5Gc-coated envelope virus from other mammalian species may represent such selective pressure. A possible scenario for the further elimination of “signal transduction-competent but sialic acid recognition-incompetent” Siglec-XII may be that the recruitment of SHP-2 by Siglec-XII (47) on epithelial cells may assist the transformation of the epithelial cell by an oncogenic driver (e.g., receptor tyrosine kinase or RAS mutation/amplification) through activation of MAPK pathway (48–52), which may have been disadvantageous for the overall fitness of the carriers of the functional allele. However, at present there is no solid experimental evidence to support these speculations.

Primate Siglec-9 (from chimpanzee, gorilla, and baboon) also prefers Neu5Gc, whereas human Siglec-9 appears to have acquired affinity toward Neu5Ac (37, 38). Human CD33/Siglec-3 and Siglec-5 also show a similar acquired affinity to Neu5Ac compared with their counterparts in baboon, which show a strong preference for Neu5Gc (38). Given that Siglec-9 has an ortholog in rodents (Siglec-E), it may play an important role in regulating innate immunity and be indispensable (although expression patterns and functions of primate Siglec-9 and rodent Siglec-E may not completely overlap (53–55)). Human Siglec-9 may have had to undergo rapid evolution to catch up with the change in the human sialome, to resume its original functionality. It is of note that the N-terminal Ig-like domain (Ig1) of great ape Siglec-9 shows much greater inter-species sequence differences than does the adjacent C2-set Ig-like domain (Ig2) (37), which is consistent with the idea that human Siglec-9 had to evolve rapidly to respond to the loss of Neu5Gc.

In fact, the CD33-related Siglec gene cluster is among the most rapidly diversifying gene families between human and chimpanzee (56), and the N-terminal Ig-like domain of CD33-related ape Siglecs is evolving faster than the other parts of the molecule (15, 37, 57). It is of interest whether the loss of Neu5Gc contributed to the accelerated evolution of human Siglecs. Assuming this is the case, we would expect that more amino acid changes have accumulated in the first Ig-like domain (Ig1) of human Siglecs than in Ig1 of chimpanzee Siglecs. In reality, the data (Table 1) do not appear to support this prediction. Although it is true that Ig1 is undergoing faster evolution than Ig2 (total human-specific changes in Ig1 and Ig2: 33.5 and 15, respectively; total chimpanzee-specific changes in Ig1 and Ig2: 40.5 and 14, respectively; average amino acid length of Ig1 and Ig2: 126 and 96, respectively), the Ig1 of Siglecs in the lineage leading to human has accumulated less sequence changes than that leading to chimpanzee. Some of the sequence changes in human Siglecs probably represent a genuine sign of selection due to the loss of Neu5Gc, whereas the majority of them may not be. Ig1 of primate Siglecs (and likely those in other species) is evolving rapidly under selective pressure that may include, but not be limited to, the changes in the landscape of endogenous sialoglycans (38).

It is of note that, in contrast to the adaptation of some human Siglecs to Neu5Ac-dominant sialome, many Siglecs still appear to prefer Neu5Gc (Table 1). Possible explanations for this fact may include: (1) the adaptation of human Siglecs to Neu5Ac-dominant sialome is still incomplete, and over the time (in the scale of millions of years) most human Siglecs will eventually acquire Neu5Ac preference; (2) some Siglecs did not have strong preference toward Neu5Gc over Neu5Ac prior to the loss of Neu5Gc in human ancestor, or have already accumulated mutations to make them sufficiently suitable for Neu5Ac recognition, thus it is not necessary for them to adapt further to Neu5Ac-dominant sialome; (3) the interaction of Siglecs with exogenous ligands (e.g., bacterial nonulosonic acids) prevent complete switch from Neu5Gc to Neu5Ac preference. Although these explanations are purely speculative, some of these scenarios may be tested experimentally. For example, an independent event has eliminated Neu5Gc in the lineage leading to New World monkeys approximately 30 million years ago (58). In contrast, the timing of Neu5Gc loss in human is far more recent, which is estimated to be 3 million years ago (26). It would be interesting to see whether the Siglecs in New World monkeys prefer Neu5Ac, or some of them still prefer Neu5Gc, to test the validity of the explanation (1) above.

## Possible Influences of Neu5Gc Loss on Human Siglecs: Altered Expression Patterns

It is of interest to know whether there is any change in the expression patterns of Siglecs between human and chimpanzee, which might also represent a consequence of Neu5Gc loss in human. Antibody-based comparative analyses of Siglec expression patterns in human and chimpanzee (and gorilla) have revealed several examples of altered expression of Siglecs in human, as summarized in Table 2. Naturally, it is more difficult to establish the influence of the loss of Neu5Gc on the expression patterns of Siglecs than its effect on the binding preferences of Siglecs, as it is indirect. Nevertheless, it appears to be implied in some cases.

**Table 2:** Expression patterns of human and chimpanzee Siglecs.

**Siglec**	**Human**	**Chimpanzee**	**References**
Sialoadhesin/Siglec-1	Mac	Mac (broader)	(39)
CD22/Siglec-2	B	B (mRNA)	(39)
CD33/Siglec-3	Mono, Mac (broader), Microglia	Mono, Mac, Microglia	(38)
MAG/Siglec-4	Schwann cells, Oligodendroglia	(Myelin)	(59, 60)
Siglec-5	Neutro, Mac (broader), B (low), **amniotic epithelium**	Neutro, Mac, **T**, B	(38, 61–63)
Siglec-6	B, DC subset, **placenta**	B	(64)
Siglec-7	NK, Mono, Mast, Neutro, Baso, Platelets, T (subset)	ND	(65–70)
Siglec-8	Eosino, Baso, Mast	ND	(71, 72)
Siglec-9	Neutro, Mono, Mac (broader)	Neutro, Mono, Mac	(38)
Siglec-10	B, Mono, DC	ND	(73, 74)
Siglec-11	Mac, **Microglia**, ovarian fibroblasts	Mac, ovarian fibroblasts	(43, 75–77)
Siglec-12	Mac, lumenal epithelia	Mac, lumenal epithelia	(36, 46)
Siglec-13	X (absent)	Mono	(45)
Siglec-14	Neutro, Mono, **amniotic epithelium**	Neutro (& Mono?)	(62, 63)
Siglec-15	OC, Mac subset	ND	(78, 79, 80, 81)
Siglec-16	Mac, **Microglia**	Mac	(43, 75, 82)

*Mac, macrophage; Mono, monocytes; B, B cells; T, T cells; NK, natural killer cells; Mast, mast cells; Neutro, neutrophils; Eosino, eosinophils; Baso, basophils; DC, dendritic cells; OC, osteoclasts; ND, not determined*.

^*^Tissue/cell type that showed clear difference in Siglec expression between human and chimpanzee are highlighted with underline and bold typeface. Reports that directly compared human and chimpanzee Siglec expression patterns are primarily cited in this table. For human Siglecs, expression in the cell types not listed in the table are also reported, such as: CD22/Siglec-2 on basophils (83); CD33/Siglec-3 on mast cells (84), basophils and neutrophils (low) (85); Siglec-6 on mast cells (86); Siglec-9 on T cell subset (68) and NK cell subset (55). Note that the expression of Siglec-6 on human B cells is restricted to CD27^+^ memory B cells (87). Tumor-infiltrating T cells express several human Siglecs, including CD33/Siglec-3, Sigec-5, Siglec-7, Siglec-9, and Siglec-10 (88).

The first reported example of altered expression of Siglec in human compared with chimpanzee was the wider distribution of Sialoadhesin/Siglec-1^+^ macrophages in chimpanzee spleen as compared with those in human spleen (39). Although the binding specificity of chimpanzee Sialoadhesin/Siglec-1 has not been analyzed, given that both human and mouse Sialoadhesin/Siglec-1 preferentially recognize Neu5Ac (39) and the sequence differences between human and chimpanzee Sialoadhesin/Siglec-1 are small (Table 1), it is likely that chimpanzee Sialoadhesin/Siglec-1 prefers Neu5Ac. Thus, the altered distribution of human Sialoadhesin/Siglec-1^+^ macrophages may be a consequence of the loss of Neu5Gc in humans (39). It is possible that the altered distribution of Sialoadhesin/Siglec-1^+^ macrophages may be more relevant to the increased density of Neu5Ac in human tissues that may influence the migration of macrophages, rather than a change in cell types that express Sialoadhesin/Siglec-1. In this regard, it would be interesting to know whether the distribution of Sialoadhesin/Siglec-1^+^ macrophages in *Cmah* knockout mice is different from that in wild-type mice.

One of the most striking changes in Siglec expression patterns in the human immune system is the near-complete absence of Siglec-5 on human T cells, in contrast to its prominent expression on chimpanzee and gorilla T cells (61, 62). The loss of Siglec-5 from human T cells appears to be correlated with the relative hyper-activation of human T cells in response to various stimuli compared with those from other great apes. [Although Siglec-5 and Siglec-14 show extremely high sequence similarity at the extracellular domain, one study (62) used a combination of antibodies that distinguish Siglec-5 and Siglec-14 to demonstrate that Siglec-5 is expressed on chimpanzee T cells]. However, it is not clear whether the loss of Siglec-5 on human T cells has a causative relationship with the loss of Neu5Gc, as human Siglec-5 does not show strong preference for either Neu5Ac or Neu5Gc (38), and its great ape counterparts have a mutation at the essential arginine residue and lack the ability to recognize sialic acids (15, 89). It is also worth mentioning that a recent work demonstrated that Siglec-5 is inducibly expressed by the activation of human T cells (88).

Siglec-11 and Siglec-16 also have undergone unique changes in their expression patterns in humans. Whereas, human Siglec-11 and Siglec-16 are expressed on brain microglia and tissue macrophages, chimpanzee Siglec-11 and Siglec-16 appear to be absent on microglia (but present on tissue macrophages) (43, 75). The change in expression patterns appears to be a consequence of a partial gene conversion of *SIGLEC11* by *SIGLEC16*. Of note, *SIGLEC16* in humans has functional and non-functional alleles (82), and the non-functional allele appears to be the one that converted *SIGLEC11* (90). *SIGLEC11* and *SIGLEC16* have undergone a complex series of concerted evolution through gene conversions in human lineage (90) and also in other lineages of apes (44). Both human and chimpanzee Siglec-11 and Siglec-16 appear to prefer Neu5Gc over Neu5Ac (43, 44), and thus it is tempting to speculate that the loss of Neu5Gc may have had some influence on the altered expression patterns of these Siglecs. Although it is known that the Neu5Gc level is extremely low in mammalian brains (91), Siglec-11 and Siglec-16 also preferentially recognize α2-8–linked Neu5Ac dimers, which are abundant in the brain and serve as ligands for these Siglecs on human microglia.

Siglec-6 was also reported to show different expression patterns between human and chimpanzee. Both human and chimpanzee Siglec-6 are expressed on B cells, whereas its expression on placental trophoblasts is observed only in humans (64). This altered expression is thought to be associated with the sequence change in the promoter region and transcription factor binding (64).

There are some reports of the presence of Siglec ligands in human tissues that are absent in chimpanzee tissues (64, 76). Although the exact nature of these ligands has not been identified, these findings imply that the difference in Siglec ligand expression patterns beyond the absence/presence of Neu5Gc may exist between human and chimpanzee and may also contribute to the rapid evolution of the Siglec family (particularly at Ig1) and/or their altered expression patterns.

## An Alternative Driving Force Behind Siglec Evolution: Interaction With Microbes

Given that Ig1 of Siglecs (particularly that of CD33-related Siglecs) is undergoing rapid evolution (57), and not all of this may be attributed to the changing endogenous ligand landscape, there is likely an alternative driving force behind their rapid evolution. Obviously, one such force could be microbial pathogens that engage Siglecs. Indeed, recent studies have provided evidence that many Siglecs are involved in the interaction with various pathogenic microbes [for recent reviews, see (92, 93)]. These microbes include viruses, bacteria, and eukaryotic pathogens (Table 3). Many of them cover themselves with sialic acids (either by *de novo* biosynthesis or by “salvage” from the human body by various mechanisms), which may be considered examples of “molecular mimicry” by microbes.

**Table 3:** Direct interaction of human Siglecs and microbes.

**Microbe**	**Microbial molecule involved**	**Human siglec involved**	**Outcome**	**References**
**BACTERIA**				
*Neisseria meningitidis*	Sialic acids on LPS	Sialoadhesin/Siglec-1 Siglec-5	Enhanced binding and phagocytosis	(94)
*Campylobacter jejuni*	Sialic acids on LPS	Sialoadhesin/Siglec-1 Siglec-7	Modulation of factors affecting helper T-cell differentiation	(95–97)
	Pseudaminic acid on flagellin	Siglec-10	Promote anti-inflammatory response	(74)
Group B Streptococcus type III	Sialic acids on CPS	Siglec-9	Attenuated immune responses	(98, 99)
Group B Streptococcus type Ia	β protein (Sia-independent)	Siglec-5 Siglec-14	Siglec-5: Attenuated responses Siglec-14: Enhanced responses	(100, 63)
		Siglec-13 (chimpanzee)	Attenuated response	(45)
*Pseudomonas aeruginosa*	Sialic acids on glycoproteins, adsorbed from human body fluid	Siglec-9	Attenuated immune responses	(101)
Non-typeable *Haemophilus influenzae*	Sialic acids on LOS + Sia-independent interaction	Siglec-5 Siglec-14	Siglec-5: Attenuated responses Siglec-14: Enhanced responses	(102)
*Escherichia coli* K1 strain	CPS (polysialic acids)	Siglec-11 Siglec-16	Siglec-11: Attenuated responses Siglec-16: Enhanced responses	(75)
**VIRUSES**				
Human immunodeficiency virus (HIV)	Sialic acids on gp120 envelope glycoprotein; host-derived gangliosides on envelope	Sialoadhesin/Siglec-1 Siglec-7	Enhanced infection	(103–108)
Varicella zoster virus (VZV), herpes simplex virus (HSV)	Glycoprotein B (sialic acids required)	MAG/Siglec-4	Enhanced infection	(109, 110)
**EUKARYOTES**				
*Candida albicans*	zymosan (?)	Siglec-7	Enhanced immune responses	(111)
*Leishmania donovani*	Surface sialic acids	Sialoadhesin/Siglec-1 Siglec-5	Enhanced infection	(112)

*Updated from Angata and Varki (93)*.

The majority of the microbes reported to interact with Siglecs so far are bacteria (Table 3). This makes sense, as sialic acids (and sialic acid-like nonulosonic acids) are occasionally found in bacterial extracellular components, such as lipopolysaccharides/lipooligosaccharides (LPS/LOS), capsular polysaccharides (CPS), and flagella. For example, group B streptococcus (GBS) type III interacts with Siglec-9 through sialylated CPS and dampens inflammatory responses by neutrophils (98), whereas GBS type Ia engages Siglec-5 by β-protein and also suppresses inflammatory responses of myeloid cells (100). It should be noted that the latter case does not involve sialic acids. Similarly, non-typeable *Haemophilus influenzae*, an opportunistic airway pathogen, engages Siglec-5 and attenuates pro-inflammatory cytokine production by myeloid cells (102), and *Escherichia coli* K1 strain, a neurotropic pathogen, engages Siglec-11 and escape killing (75). Siglecs are likely under pressure to escape the exploitation by these pathogens, which may partially explain the driving force behind their rapid evolution.

It appears that Siglecs were not just escaping from these pathogens; they appear to have developed “counter-traps” against these pathogens. Some Siglecs (i.e., Siglec-5 and Siglec-14; Siglec-11 and Siglec-16) are found to be “paired receptors,” which are two Siglecs with highly homologous extracellular domains recognizing similar ligands, combined with intracellular signaling modules transducing opposing signals (i.e., one of the pair interacts with SHP-1 and transduces the inhibitory signal, whereas the other interacts with adapter protein DAP12 and tyrosine kinase Syk and transduces the activating signal). In fact, whereas the engagement of inhibitory Siglec by pathogenic bacteria suppresses anti-bacterial responses, the engagement of activating Siglec counteracts this effect (75, 102, 63). It is of note that these “paired” Siglecs appear to show more sequence differences between human and chimpanzee than other “stand-alone” Siglecs (Table 1), possibly implying that these Siglecs are under higher selective pressure to diversify than are other Siglecs. These paired Siglecs are undergoing concerted evolution through repeated gene conversions (43, 44, 89, 90), which is likely necessary to maintain the effectiveness of activating-type Siglec as “counter-traps.” It is also intriguing that, in humans, null alleles for these activating-type Siglecs (Siglec-14 and Siglec-16) are found at very high frequencies (82, 113).

Evidence supporting the relevance of these interactions between Siglecs and bacterial pathogens in infectious diseases is emerging from genetic association studies (Table 4). Small-scale case–control studies investigating the possible correlations between the polymorphisms of *SIGLEC* genes and infectious disease susceptibility have revealed some correlations, such as *SIGLEC14* null polymorphism and COPD exacerbation (102), pre-term delivery in the presence of GBS (63), *Mycobacterium tuberculosis* meningitis (138), and *SIGLEC9* polymorphism and COPD exacerbation (133). In addition, large-scale genome-wide association studies (GWAS) have also revealed possible associations between *SIGLEC* polymorphisms and infectious diseases, such as *SIGLEC5* polymorphism and leprosy (129) and severe periodontitis (128), although these GWAS did not demonstrate a direct interaction between the etiological agents and Siglec protein. Some *SIGLEC* genetic polymorphisms appear to influence the leukocyte counts (142); thus it is possible that the influence of *SIGLEC* genetic polymorphisms on antibacterial defense may be indirect. Regardless, the application of GWAS to infectious diseases may further reveal the relevance of Siglecs for immunological defense against bacterial pathogens.

**Table 4:** Polymorphisms in human *SIGLEC* genes and association with disease/phenotype.

**Gene**	**Polymorphism**	**Associated phenotype**	**References**
*SIGLEC1*	rs656635, rs609203, rs3859664, rs4813636 (SNPs in intron or 3'UTR)	Lung function	(114)
*SIGLEC1*	rs6037651 (nonsynonymous SNP)	Serum IgM level	(115)
*CD22*	rs34826052 (synonymous SNP)	Limited cutaneous systemic sclerosis	(116)
*CD22*	rs4805119 etc. (intronic SNP)	B-precursor leukemia	(117, 118)
*CD33*	rs3865444 (promoter SNP) rs12459419 (nonsynonymous SNP, influencing splicing)	Late-onset Alzheimer's disease	(119–122)
*CD33*	rs35112940, rs12459419 (nonsynonymous SNPs)	Efficiency of antibody therapy in pediatric acute myeloid leukemia	(123, 124)
*MAG*	rs720309 (intronic SNP) rs7249617 (intronic SNP)	Schizophrenia	(125, 126) (127)
*SIGLEC5*	rs4284742 (intronic SNP)	Periodontitis	(128)
*SIGLEC5*	rs10414149 (intronic SNP)	Leprosy	(129)
*SIGLEC6*	rs2305772 (non-synonymous SNP, influencing splicing)	Systemic lupus erythematosus	(130)
*SIGLEC8*	rs36498 (promoter SNP) rs10409962 (nonsynonymous SNP)	Allergic asthma	(131)
*SIGLEC9*	rs16988910 (nonsynonymous SNP)	Short-term survival of lung cancer patients; Emphysema	(132)
*SIGLEC9*	rs2075803, rs2258983 (nonsynonymous SNP)	COPD exacerbation	(133)
*SIGLEC11*	rs12165127 (intronic SNP)	Lung cancer in never-smokers	(134)
*SIGLEC12*	rs16982743 (stop codon generated)	Cardiovascular outcomes in patients with hypertension on antihypertensive therapy	(135)
*SIGLEC12*	rs3752135 (nonsynonymous SNP)	Stress fracture	(136)
*SIGLEC14*	rs10412972, rs11084102 (upstream SNPs)	Plasma plasminogen level	(137)
*SIGLEC14*	*SIGLEC14-SIGLEC5* fusion (*SIGLEC14* deletion)	COPD exacerbation	(102)
*SIGLEC14*	*SIGLEC14-SIGLEC5* fusion (*SIGLEC14* deletion)	Pre-term delivery in the presence of GBS infection	(63)
*SIGLEC14*	*SIGLEC14-SIGLEC5* fusion (*SIGLEC14* deletion)	*Mycobacterium tuberculosis* meningitis	(138)
Various	Various	Plasma protein levels	(139, 140)
Various	Various	Cerebrospinal fluid protein levels	(141)
Various	Various	Blood cell counts	(142)

*Updated from Angata (16) and Angata (143)*.

*Some of the studies listed above are small-scale case–control studies, whereas some others are large-scale genome-wide association studies (GWAS). Some of the associations listed are not prominently featured in the references cited but found in the GWAS catalog (https://www.ebi.ac.uk/gwas/)*.

With regard to viral pathogens, recent studies have revealed that Sialoadhesin/Siglec-1 (also known as CD169) may play a major role in retrovirus infection (103). For example, several groups have reported that human immunodeficiency virus (HIV) exploits Sialoadhesin/Siglec-1 to enhance infection of CD4^+^ T cells (the primary target cells) by trans-infection (i.e., the virus particle is captured by macrophages with Sialoadhesin/Siglec-1, which transfers the virus to CD4^+^ T cells and facilitates the infection) (104–107). Although a rare “null” mutation in the *SIGLEC1* gene was found not to protect carriers from HIV infection (144), the low frequency of this mutation (allele frequency: ~1.3% in Europeans) may preclude us from making a definitive conclusion (145). Given that Sialoadhesin/Siglec-1 appears to be involved in retroviral infection in both mouse and human (103), one may expect that it should evolve rapidly to avoid viral infections; however, Sialoadhesin/Siglec-1 does not appear to be evolving rapidly (Table 1). This may be because enveloped viruses are coated with a host-derived membrane (a part of “self”), and thus there is no way Sialoadhesin/Siglec-1 can evolve to completely evade such an interaction (unless the virus develops a protein that binds Sialoadhesin/Siglec-1 in sialic acid-independent manner). It is worth noting that myelin-associated glycoprotein (MAG)/Siglec-4, the other Siglec known to interact with another enveloped virus (109, 110), is also highly conserved among mammals, and in both cases sialic acids are required for the interaction between the virus and Siglecs (Table 3).

## Conclusion and Perspectives

*Cmah* null mouse is a valuable tool for the investigation of the physiological roles of Neu5Gc and the short-term consequences of its loss, although it may not be a perfect model of modern human. Using this mouse model, it was shown that the expression of Neu5Gc itself makes T cells less responsive to stimulus, without any change in Siglec expression (146). Likewise, Neu5Gc appears to have a general suppressive effect on mouse monocyte/macrophage activities, without the apparent involvement of Siglecs (147). In line with these findings, the loss of Neu5Gc has had major influences on human biology that reach far beyond Siglecs (148), explaining some of the differences between human and our close relatives (e.g., chimpanzee) in pathophysiological phenotypes (149). Although Neu5Gc from dietary sources (in the form of meat or milk from the animals that express Neu5Gc) can be incorporated into human tissue glycoproteins and glycolipids (150), the level of Neu5Gc in human tissues tends to be low, accounting for <1% of total sialic acids (151). Given that the current set of human Siglecs lack a strong preference toward Neu5Gc, and the affinity between Siglecs and sialic acids tends to be low (*K*_d_ in ~mM range), this level of Neu5Gc in human tissues may not influence human physiology by way of Siglecs. The trace amount of Neu5Gc incorporated into human tissues may be more relevant to the bacterial toxins that specifically recognize Neu5Gc (152) and xeno-autoantibodies that recognize Neu5Gc (as discussed in other articles of this series). Regardless, the loss of Neu5Gc appears to have left some footprint on the evolution of human Siglecs, as discussed above.

The evolution of human Siglecs was also likely influenced by the interaction with microbes. A recent population genetics-based study implied that some Siglecs may have been subjected to population-specific hard selective sweeps, as judged by the presence of long-range linkage disequilibrium (153). These *SIGLEC* genes include *SIGLEC8* and *SIGLEC10* among Africans, *SIGLEC5, SIGLEC6, SIGLEC12*, and *SIGLEC14* among Europeans, and *CD22* and *MAG* among Asians. Although it remains speculative, the population-specific difference in the signatures of selection imply that the evolution of the Siglec family in the human population is an ongoing process, and different pathogen pressures are present in different geographical locations (or through different agricultural constraints, e.g., use of different domestic animals, which may carry different kinds of bacteria/viruses).

Many questions remain with regard to the function and evolution of Siglecs. For example, do viruses really target only conserved Siglecs and are they not relevant to the rapid evolution of Siglecs? What was or is the selective force behind the spread of “null” alleles of *SIGLEC14* and *SIGLEC16* (and perhaps others, such as *SIGLEC1*) in modern human populations? Do the bacteria that express sialic acid-like nonulosonic acids (154) generally engage Siglecs to modulate immune responses and thus play a role in the evolution of Siglecs? Does the interaction between Siglecs and commensal bacteria (e.g., normal gut microbiota) play any role in the modulation of immunity and the evolution of Siglecs? Some of these questions can be addressed experimentally and will deepen our understanding of the biology of Siglecs and sialic acids.

## Author Contributions

TA analyzed the literature and wrote the manuscript.

### Conflict of Interest Statement

The author declares that the research was conducted in the absence of any commercial or financial relationships that could be construed as a potential conflict of interest.

## References

[1] Kawai T Akira S. Toll-like receptors and their crosstalk with other innate receptors in infection and immunity. Immunity. (2011) 34:637–50. 10.1016/j.immuni.2011.05.006 21616434

[2] Geijtenbeek T Gringhuis S. Signalling through C-type lectin receptors: shaping immune responses. Nat Rev Immunol. (2009) 9:465–79. 10.1038/nri2569 19521399 PMC7097056

[3] Yoneyama M Onomoto K Jogi M Akaboshi T Fujita T. Viral RNA detection by RIG-I-like receptors. Curr Opin Immunol. (2015) 32:48–53. 10.1016/j.coi.2014.12.012 25594890

[4] Kanneganti TD Lamkanfi M Nunez G. Intracellular NOD-like receptors in host defense and disease. Immunity (2007) 27:549–59. 10.1016/j.immuni.2007.10.002 17967410

[5] Varki A. Since there are PAMPs and DAMPs, there must be SAMPs? Glycan “self-associated molecular patterns” dampen innate immunity, but pathogens can mimic them. Glycobiology (2011) 21:1121–4. 10.1093/glycob/cwr087 21932452 PMC3150115

[6] Medzhitov R Janeway CA Jr. Decoding the patterns of self and nonself by the innate immune system. Science (2002) 296:298–300. 10.1126/science.1068883 11951031

[7] Angata T Varki A. Chemical diversity in the sialic acids and related alpha-keto acids: an evolutionary perspective. Chem Rev. (2002) 102:439–70. 10.1021/cr000407m 11841250

[8] Varki A Angata T. Siglecs - the major subfamily of I-type lectins. Glycobiology (2006) 16:1R−27R. 10.1093/glycob/cwj008 16014749

[9] Crocker P Paulson J Varki A. Siglecs and their roles in the immune system. Nat Rev Immunol. (2007) 7:255–66. 10.1038/nri2056 17380156

[10] Pillai S Netravali I Cariappa A Mattoo H. Siglecs and immune regulation. Annu Rev Immunol. (2012) 30:357–92. 10.1146/annurev-immunol-020711-075018 22224769 PMC3781015

[11] Macauley M Crocker P Paulson J. Siglec-mediated regulation of immune cell function in disease. Nat Rev Immunol. (2014) 14:653–66. 10.1038/nri3737 25234143 PMC4191907

[12] Crocker P Clark E Filbin M Gordon S Jones Y Kehrl J. Siglecs: a family of sialic-acid binding lectins [letter]. Glycobiology (1998) 8:v. 10.1093/oxfordjournals.glycob.a018832 9498912

[13] Angata T Varki A. Cloning, characterization, and phylogenetic analysis of siglec-9, a new member of the CD33-related group of siglecs. Evidence for co-evolution with sialic acid synthesis pathways. J Biol Chem. (2000) 275:22127–35. 10.1074/jbc.M002775200 10801860

[14] Bornhofft KF Goldammer T Rebl A Galuska SP. Siglecs: a journey through the evolution of sialic acid-binding immunoglobulin-type lectins. Dev Comp Immunol. (2018) 86:219–31. 10.1016/j.dci.2018.05.008 29751010

[15] Angata T Margulies E Green E Varki A. Large-scale sequencing of the CD33-related Siglec gene cluster in five mammalian species reveals rapid evolution by multiple mechanisms. Proc Natl Acad Sci USA. (2004) 101:13251–6. 10.1073/pnas.0404833101 15331780 PMC516556

[16] Angata T Polymorphisms and mutations in SIGLEC genes and their associations with diseases. J Japan Biochem Soc. (2017) 89:652–9. 10.14952/SEIKAGAKU.2017.890652

[17] Beatson R Tajadura-Ortega V Achkova D Picco G Tsourouktsoglou TD Klausing S. The mucin MUC1 modulates the tumor immunological microenvironment through engagement of the lectin Siglec-9. Nat Immunol. (2016) 17:1273–81. 10.1038/ni.3552 27595232 PMC5257269

[18] Carroll DJ O'Sullivan JA Nix DB Cao Y Tiemeyer M Bochner BS. Sialic acid-binding immunoglobulin-like lectin 8 (Siglec-8) is an activating receptor mediating beta2-integrin-dependent function in human eosinophils. J Allergy Clin Immunol. (2018) 141:2196–207. 10.1016/j.jaci.2017.08.013 28888781 PMC5839929

[19] Brown D Trowsdale J Allen R. The LILR family: modulators of innate and adaptive immune pathways in health and disease. Tissue Antigens (2004) 64:215–25. 10.1111/j.0001-2815.2004.00290.x 15304001

[20] Parham P Guethlein LA. Genetics of natural killer cells in human health, disease, and survival. Annu Rev Immunol. (2018) 36:519–48. 10.1146/annurev-immunol-042617-053149 29394121

[21] Trowsdale J. Genetic and functional relationships between MHC and NK receptor genes. Immunity (2001) 15:363–74. 10.1016/S1074-7613(01)00197-2 11567627

[22] Varki A Schnaar RL Schauer R Sialic Acids and other nonulosonic acids. In: Varki A Cummings RD Esko JD Stanley P Hart GW editors. Essentials of Glycobiology. 3rd ed. New York, NY: Cold Spring Harbor; Laboratory Press (2015). pp. 179–95.

[23] Chou H Takematsu H Diaz S Iber J Nickerson E Wright K. A mutation in human CMP-sialic acid hydroxylase occurred after the Homo-Pan divergence. Proc Natl Acad Sci USA. (1998) 95:11751–6. 10.1073/pnas.95.20.11751 9751737 PMC21712

[24] Irie A Koyama S Kozutsumi Y Kawasaki T Suzuki A. The molecular basis for the absence of N-glycolylneuraminic acid in humans. J Biol Chem. (1998) 273:15866–71. 10.1074/jbc.273.25.15866 9624188

[25] Hayakawa T Satta Y Gagneux P Varki A Takahata N. Alu-mediated inactivation of the human CMP- N-acetylneuraminic acid hydroxylase gene. Proc Natl Acad Sci USA. (2001) 98:11399–404. 10.1073/pnas.191268198 11562455 PMC58741

[26] Chou H Hayakawa T Diaz S Krings M Indriati E Leakey M. Inactivation of CMP-N-acetylneuraminic acid hydroxylase occurred prior to brain expansion during human evolution. Proc Natl Acad Sci USA. (2002) 99:11736–41. 10.1073/pnas.182257399 12192086 PMC129338

[27] Varki A. Multiple changes in sialic acid biology during human evolution. Glycoconj J. (2009) 26:231–45. 10.1007/s10719-008-9183-z 18777136 PMC7087641

[28] Peri S Kulkarni A Feyertag F Berninsone PM Alvarez-Ponce D. Phylogenetic distribution of CMP-Neu5Ac hydroxylase (CMAH), the enzyme synthetizing the proinflammatory human xenoantigen Neu5Gc. Genome Biol Evol. (2018) 10:207–19. 10.1093/gbe/evx251 29206915 PMC5767959

[29] Kelm S Schauer R Manuguerra JC Gross HJ Crocker PR. Modifications of cell surface sialic acids modulate cell adhesion mediated by sialoadhesin and CD22. Glycoconj J. (1994) 11:576–85. 10.1007/BF00731309 7696861

[30] Naito Y Takematsu H Koyama S Miyake S Yamamoto H Fujinawa R. Germinal center marker GL7 probes activation-dependent repression of N-glycolylneuraminic acid, a sialic acid species involved in the negative modulation of B-cell activation. Mol Cell Biol. (2007) 27:3008–22. 10.1128/MCB.02047-06 17296732 PMC1899932

[31] Macauley MS Kawasaki N Peng W Wang SH He Y Arlian BM. Unmasking of CD22 co-receptor on germinal center b-cells occurs by alternative mechanisms in mouse and man. J Biol Chem. (2015) 290:30066–77. 10.1074/jbc.M115.691337 26507663 PMC4705971

[32] Duong BH Tian H Ota T Completo G Han S Vela JL. Decoration of T-independent antigen with ligands for CD22 and Siglec-G can suppress immunity and induce B cell tolerance *in vivo*. J Exp Med. (2010) 207:173–87. 10.1084/jem.20091873 20038598 PMC2812539

[33] Collins B Kiso M Hasegawa A Tropak M Roder J Crocker P. Binding specificities of the sialoadhesin family of I-type lectins - Sialic acid linkage and substructure requirements for binding of myelin-associated glycoprotein, Schwann cell myelin protein, and sialoadhesin. J Biol Chem. (1997) 272:16889–95. 10.1074/jbc.272.27.16889 9201997

[34] Collins B Yang L Mukhopadhyay G Filbin M Kiso M Hasegawa A. Sialic acid specificity of myelin-associated glycoprotein binding. J Biol Chem. (1997) 272:1248–55. 10.1074/jbc.272.2.1248 8995428

[35] Brinkman-Van der Linden E Varki A New aspects of siglec binding specificities, including the significance of fucosylation and of the sialyl-Tn epitope. J Biol Chem. (2000) 275:8625–32. 10.1074/jbc.275.12.8625 10722702

[36] Angata T Varki N Varki A. A second uniquely human mutation affecting sialic acid biology. J Biol Chem. (2001) 276:40282–7. 10.1074/jbc.M105926200 11546777

[37] Sonnenburg J Altheide T Varki A. A uniquely human consequence of domain-specific functional adaptation in a sialic acid-binding receptor. Glycobiology (2004) 14:339–46. 10.1093/glycob/cwh039 14693915

[38] Padler-Karavani V Hurtado-Ziola N Chang Y Sonnenburg J Ronaghy A Yu H. Rapid evolution of binding specificities and expression patterns of inhibitory CD33-related Siglecs in primates. FASEB J. (2014) 28:1280–93. 10.1096/fj.13-241497 24308974 PMC3929681

[39] Brinkman-Van der Linden E Sjoberg E Juneja L Crocker P Varki N Varki A Loss of N-glycolylneuraminic acid in human evolution - Implications for sialic acid recognition by siglecs. J Biol Chem. (2000) 275:8633–40. 10.1074/jbc.275.12.8633 10722703

[40] Blixt O Collins B Van Den Nieuwenhof I Crocker P Paulson J. Sialoside specificity of the Siglec family assessed using novel multivalent probes: Identification of potent inhibitors of myelin associated glycoprotein. J Biol Chem. (2003) 278 31007–19. 10.1074/jbc.M304331200 12773526

[41] Collins B Ito H Sawada N Ishida H Kiso M Schnaar R. Enhanced binding of the neural siglecs, myelin-associated glycoprotein and Schwann cell myelin protein, to Chol-1 (alpha-series) gangliosides and novel sulfated Chol-1 analogs. J Biol Chem. (1999) 274:37637–43. 10.1074/jbc.274.53.37637 10608819

[42] Bochner B Alvarez R Mehta P Bovin N Blixt O White J. Glycan array screening reveals a candidate ligand for Siglec-8. J Biol Chem. (2005) 280:4307–12. 10.1074/jbc.M412378200 15563466

[43] Hayakawa T Angata T Lewis A Mikkelsen T Varki N Varki A. A human-specific gene in microglia. Science. (2005) 309:1693. 10.1126/science.1114321 16151003

[44] Hayakawa T Khedri Z Schwarz F Landig C Liang SY Yu H. Coevolution of Siglec−11 and Siglec−16 via gene conversion in primates. BMC Evol Biol. (2017) 17:228. 10.1186/s12862-017-1075-z 29169316 PMC5701461

[45] Wang X Mitra N Secundino I Banda K Cruz P Padler-Karavani V. Specific inactivation of two immunomodulatory SIGLEC genes during human evolution. Proc Natl Acad Sci USA. (2012) 109:9935–40. 10.1073/pnas.1119459109 22665810 PMC3382539

[46] Mitra N Banda K Altheide T Schaffer L Johnson-Pais T Beuten J. SIGLEC12, a human-specific segregating (pseudo)gene, encodes a signaling molecule expressed in prostate carcinomas. J Biol Chem. (2011) 286:23003–11. 10.1074/jbc.M111.244152 21555517 PMC3123068

[47] Yu Z Lai C Maoui M Banville D Shen S. Identification and characterization of S2V, a novel putative siglec that contains two V set Ig-like domains and recruits protein-tyrosine phosphatases SHPs. J Biol Chem. (2001) 276:23816–24. 10.1074/jbc.M102394200 11328818

[48] Dardaei L Wang HQ Singh M Fordjour P Shaw KX Yoda S. SHP2 inhibition restores sensitivity in ALK-rearranged non-small-cell lung cancer resistant to ALK inhibitors. Nat Med. (2018) 24:512–7. 10.1038/nm.4497 29505033 PMC6343825

[49] Mainardi S Mulero-Sanchez A Prahallad A Germano G Bosma A Krimpenfort P. SHP2 is required for growth of KRAS-mutant non-small-cell lung cancer *in vivo*. Nat Med. (2018) 24:961–7. 10.1038/s41591-018-0023-9 29808006

[50] Ruess DA Heynen GJ Ciecielski KJ Ai J Berninger A Kabacaoglu D. Mutant KRAS-driven cancers depend on PTPN11/SHP2 phosphatase. Nat Med. (2018) 24:954–60. 10.1038/s41591-018-0024-8 29808009

[51] Wong GS Zhou J Liu JB Wu Z Xu X Li T Targeting wild-type KRAS-amplified gastroesophageal cancer through combined MEK and SHP2 inhibition. Nat Med. (2018) 24:968–77. 10.1038/s41591-018-0022-x 29808010 PMC6039276

[52] Nichols RJ Haderk F Stahlhut C Schulze CJ Hemmati G Wildes D. RAS nucleotide cycling underlies the SHP2 phosphatase dependence of mutant BRAF-, NF1- and RAS-driven cancers. Nat Cell Biol. (2018) 20:1064–73. 10.1038/s41556-018-0169-1 30104724 PMC6115280

[53] Zhang J Biedermann B Nitschke L Crocker P. The murine inhibitory receptor mSiglec-E is expressed broadly on cells of the innate immune system whereas mSiglec-F is restricted to eosinophils. Eur J Immunol. (2004) 34:1175–84. 10.1002/eji.200324723 15048729

[54] von Gunten S Yousefi S Seitz M Jakob S Schaffner T Seger R Siglec-9 transduces apoptotic and nonapoptotic death signals into neutrophils depending on the proinflammatory cytokine environment. Blood (2005) 106:1423–31. 10.1182/blood-2004-10-4112 15827126

[55] Jandus C Boligan K Chijioke O Liu H Dahlhaus M Demoulins T. Interactions between Siglec-7/9 receptors and ligands influence NK cell-dependent tumor immunosurveillance. J Clin Invest. (2014) 124:1810–20. 10.1172/JCI65899 24569453 PMC3973073

[56] The Chimpanzee Sequencing and Analysis Consortium Initial Sequence of the Chimpanzee Genome and Comparison with the Human Genome. Nature. (2005) 437:69–87. 10.1038/nature04072 16136131

[57] Altheide TK Hayakawa T Mikkelsen TS Diaz S Varki N Varki A. System-wide genomic and biochemical comparisons of sialic acid biology among primates and rodents: Evidence for two modes of rapid evolution. J Biol Chem. (2006) 281:25689–702. 10.1074/jbc.M604221200 16769723

[58] Springer SA Diaz SL Gagneux P. Parallel evolution of a self-signal: humans and new world monkeys independently lost the cell surface sugar Neu5Gc. Immunogenetics (2014) 66:671–4. 10.1007/s00251-014-0795-0 25124893 PMC4198446

[59] Moller JR. Rapid conversion of myelin-associated glycoprotein to a soluble derivative in primates. Brain Res. (1996) 741:27–31. 10.1016/S0006-8993(96)00882-7 9001700

[60] Miller DJ Duka T Stimpson CD Schapiro SJ Baze WB McArthur MJ. Prolonged myelination in human neocortical evolution. Proc Natl Acad Sci USA. (2012) 109:16480–5. 10.1073/pnas.1117943109 23012402 PMC3478650

[61] Nguyen D Hurtado-Ziola N Gagneux P Varki A. Loss of Siglec expression on T lymphocytes during human evolution. Proc Natl Acad Sci USA. (2006) 103:7765–70. 10.1073/pnas.0510484103 16682635 PMC1472519

[62] Soto PC Stein LL Hurtado-Ziola N Hedrick SM Varki A. Relative over-reactivity of human versus chimpanzee lymphocytes: implications for the human diseases associated with immune activation. J Immunol. (2010) 184:4185–95. 10.4049/jimmunol.0903420 20231688 PMC3085894

[63] Ali S Fong J Carlin A Busch T Linden R Angata T. Siglec-5 and Siglec−14 are polymorphic paired receptors that modulate neutrophil and amnion signaling responses to group B Streptococcus. J Exp Med. (2014) 211:1231–42. 10.1084/jem.20131853 24799499 PMC4042635

[64] Brinkman-Van der Linden EC Hurtado-Ziola N Hayakawa T Wiggleton L Benirschke K Varki A Human-specific expression of Siglec-6 in the placenta. Glycobiology (2007) 17:922–31. 10.1093/glycob/cwm065 17580316

[65] Falco M Biassoni R Bottino C Vitale M Sivori S Augugliaro R. Identification and molecular cloning of p75/AIRM1, a novel member of the sialoadhesin family that functions as an inhibitory receptor in human natural killer cells. J Exp Med. (1999) 190:793–801. 10.1084/jem.190.6.793 10499918 PMC2195632

[66] Nicoll G Ni J Liu D Klenerman P Munday J Dubock S. Identification and characterization of a novel siglec, siglec-7, expressed by human natural killer cells and monocytes. J Biol Chem. (1999) 274:34089–95. 10.1074/jbc.274.48.34089 10567377

[67] Vitale C Romagnani C Falco M Ponte M Vitale M Moretta A Engagement of p75/AIRM1 or CD33 inhibits the proliferation of normal or leukemic myeloid cells. Proc Natl Acad Sci USA. (1999) 96:15091–6. 10.1073/pnas.96.26.15091 10611343 PMC24778

[68] Ikehara Y Ikehara S Paulson J. Negative regulation of T cell receptor signaling by Siglec-7 (p70/AIRM) and Siglec-9. J Biol Chem. (2004) 279:43117–25. 10.1074/jbc.M403538200 15292262

[69] Mizrahi S Gibbs B Karra L Ben-Zimra M Levi-Schaffer F. Siglec-7 is an inhibitory receptor on human mast cells and basophils. J Allergy Clin Immunol. (2014) 134:230–3. 10.1016/j.jaci.2014.03.031 24810846

[70] Nguyen K Hamzeh-Cognasse H Palle S Anselme-Bertrand I Arthaud C Chavarin P. Role of Siglec-7 in apoptosis in human platelets. PLoS One. (2014) 9:e106239. 10.1371/journal.pone.0106239 25230315 PMC4167548

[71] Floyd H Ni J Cornish A Zeng Z Liu D Carter K. Siglec-8. A novel eosinophil-specific member of the immunoglobulin superfamily. J Biol Chem. (2000) 275:861–6. 10.1074/jbc.275.2.861 10625619

[72] Kikly K Bochner B Freeman S Tan K Gallagher K D'alessio K. Identification of SAF-2, a novel siglec expressed on eosinophils, mast cells, and basophils. J Allergy Clin Immunol. (2000) 105(6 Pt 1):1093–100. 10.1067/mai.2000.107127 10856141

[73] Munday J Kerr S Ni J Cornish AL Zhang JQ Nicoll G. Identification, characterization and leucocyte expression of Siglec−10, a novel human sialic acid-binding receptor. Biochem J. (2001) 355(Pt 2):489–97. 10.1042/bj3550489 11284738 PMC1221762

[74] Stephenson HN Mills DC Jones H Milioris E Copland A Dorrell N. Pseudaminic acid on Campylobacter jejuni flagella modulates dendritic cell IL−10 expression via Siglec−10 receptor: a novel flagellin-host interaction. J Infect Dis. (2014) 210:1487–98. 10.1093/infdis/jiu287 24823621 PMC4195440

[75] Schwarz F Landig CS Siddiqui S Secundino I Olson J Varki N. Paired Siglec receptors generate opposite inflammatory responses to a human-specific pathogen. EMBO J. (2017) 36:751–60. 10.15252/embj.201695581 28100677 PMC5350563

[76] Wang X Chow R Deng L Anderson D Weidner N Godwin A. Expression of Siglec−11 by human and chimpanzee ovarian stromal cells, with uniquely human ligands: implications for human ovarian physiology and pathology. Glycobiology (2011) 21:1038–48. 10.1093/glycob/cwr039 21467073 PMC3130538

[77] Angata T Kerr S Greaves D Varki N Crocker P Varki A. Cloning and characterization of human Siglec−11. A recently evolved signaling molecule that can interact with SHP-1 and SHP-2 and is expressed by tissue macrophages, including brain microglia. J Biol Chem. (2002) 277:24466–74. 10.1074/jbc.M202833200 11986327

[78] Angata T Tabuchi Y Nakamura K Nakamura M. Siglec−15: an immune system Siglec conserved throughout vertebrate evolution. Glycobiology (2007) 17:838–46. 10.1093/glycob/cwm049 17483134

[79] Hiruma Y Hirai T Tsuda E. Siglec−15, a member of the sialic acid-binding lectin, is a novel regulator for osteoclast differentiation. Biochem Biophys Res Commun. (2011) 409:424–9. 10.1016/j.bbrc.2011.05.015 21586272

[80] Kameda Y Takahata M Komatsu M Mikuni S Hatakeyama S Shimizu T. Siglec−15 regulates osteoclast differentiation by modulating RANKL-induced phosphatidylinositol 3-kinase/Akt and Erk pathways in association with signaling Adaptor DAP12. J Bone Miner Res. (2013) 28:2463–75. 10.1002/jbmr.1989 23677868

[81] Takamiya R Ohtsubo K Takamatsu S Taniguchi N Angata T. The interaction between Siglec−15 and tumor-associated sialyl-Tn antigen enhances TGF-beta secretion from monocytes/macrophages through the DAP12-Syk pathway. Glycobiology (2013) 23:178–87. 10.1093/glycob/cws139 23035012

[82] Cao H Lakner U de Bono B Traherne J Trowsdale J Barrow A. SIGLEC16 encodes a DAP12-associated receptor expressed in macrophages that evolved from its inhibitory counterpart SIGLEC11 and has functional and non-functional alleles in humans. Eur J Immunol. (2008) 38:2303–15. 10.1002/eji.200738078 18629938

[83] Han K Kim Y Lee J Lim J Lee KY Kang CS. Human basophils express CD22 without expression of CD19. Cytometry (1999) 37:178–83. 10.1002/(SICI)1097-0320(19991101)37:3<178::AID-CYTO3>3.0.CO;2-Z 10520197

[84] Valent P Ashman LK Hinterberger W Eckersberger F Majdic O Lechner K. Mast cell typing: demonstration of a distinct hematopoietic cell type and evidence for immunophenotypic relationship to mononuclear phagocytes. Blood (1989) 73:1778–85. 2469499

[85] Stain C Stockinger H Scharf M Jager U Gossinger H Lechner K. Human blood basophils display a unique phenotype including activation linked membrane structures. Blood. (1987) 70:1872–9. 3118989

[86] Yokoi H Myers A Matsumoto K Crocker P Saito H Bochner B. Alteration and acquisition of Siglecs during *in vitro* maturation of CD34+ progenitors into human mast cells. Allergy (2006) 61:769–76. 10.1111/j.1398-9995.2006.01133.x 16677248

[87] Kardava L Moir S Wang W Ho J Buckner CM Posada JG. Attenuation of HIV-associated human B cell exhaustion by siRNA downregulation of inhibitory receptors. J Clin Invest. (2011) 121:2614–24. 10.1172/JCI45685 21633172 PMC3127436

[88] Stanczak MA Siddiqui SS Trefny MP Thommen DS Boligan KF von Gunten S. Self-associated molecular patterns mediate cancer immune evasion by engaging Siglecs on T cells. J Clin Invest. (2018) 128:4912–23. 10.1172/JCI120612 30130255 PMC6205408

[89] Angata T Hayakawa T Yamanaka M Varki A Nakamura M. Discovery of Siglec−14, a novel sialic acid receptor undergoing concerted evolution with Siglec-5 in primates. FASEB J. (2006) 20:1964–73. 10.1096/fj.06-5800com 17012248

[90] Wang X Mitra N Cruz P Deng L Varki N Angata T. Evolution of siglec−11 and siglec−16 genes in hominins. Mol Biol Evol. (2012) 29:2073–86. 10.1093/molbev/mss077 22383531 PMC3408085

[91] Davies LR Varki A. Why is N-glycolylneuraminic acid rare in the vertebrate brain? Top Curr Chem. (2015) 366:31–54. 10.1007/128_2013_419 23471785 PMC4026345

[92] Chang YC Nizet V. The interplay between Siglecs and sialylated pathogens. Glycobiology (2014) 24:818–25. 10.1093/glycob/cwu067 24996821 PMC4168292

[93] Angata T Varki A Siglec interactions with pathogens. In: Taniguchi N Endo T Hart GW Seeberger PH Wong C-H editors. Glycoscience: Biology and Medicine. (Tokyo: Springer) (2015). pp. 633–42.

[94] Jones C Virji M Crocker P. Recognition of sialylated meningococcal lipopolysaccharide by siglecs expressed on myeloid cells leads to enhanced bacterial uptake. Mol Microbiol. (2003) 49:1213–25. 10.1046/j.1365-2958.2003.03634.x 12940982

[95] Avril T Wagner E Willison H Crocker P. Sialic acid-binding immunoglobulin-like lectin 7 mediates selective recognition of sialylated glycans expressed on Campylobacter jejuni lipooligosaccharides. Infect Immun. (2006) 74:4133–41. 10.1128/IAI.02094-05 16790787 PMC1489752

[96] Heikema A Bergman M Richards H Crocker P Gilbert M Samsom J. Characterization of the specific interaction between sialoadhesin and sialylated Campylobacter jejuni lipooligosaccharides. Infect Immun. (2010) 78:3237–46. 10.1128/IAI.01273-09 20421384 PMC2897406

[97] Bax M Kuijf M Heikema A van Rijs W Bruijns S Garcia-Vallejo J. Campylobacter jejuni lipooligosaccharides modulate dendritic cell-mediated T cell polarization in a sialic acid linkage-dependent manner. Infect Immun. (2011) 79:2681–9. 10.1128/IAI.00009-11 21502591 PMC3191980

[98] Carlin A Uchiyama S Chang Y Lewis A Nizet V Varki A. Molecular mimicry of host sialylated glycans allows a bacterial pathogen to engage neutrophil Siglec-9 and dampen the innate immune response. Blood (2009) 113:3333–6. 10.1182/blood-2008-11-187302 19196661 PMC2665898

[99] Carlin A Lewis A Varki A Nizet V. Group B streptococcal capsular sialic acids interact with siglecs (immunoglobulin-like lectins) on human leukocytes. J Bacteriol. (2007) 189:1231–7. 10.1128/JB.01155-06 16997964 PMC1797352

[100] Carlin A Chang Y Areschoug T Lindahl G Hurtado-Ziola N King C. Group B Streptococcus suppression of phagocyte functions by protein-mediated engagement of human Siglec-5. J Exp Med. (2009) 206:1691–9. 10.1084/jem.20090691 19596804 PMC2722167

[101] Khatua B Bhattacharya K Mandal C. Sialoglycoproteins adsorbed by Pseudomonas aeruginosa facilitate their survival by impeding neutrophil extracellular trap through siglec-9. J Leukoc Biol. (2012) 91:641–55. 10.1189/jlb.0511260 22241833

[102] Angata T Ishii T Motegi T Oka R Taylor R Soto P. Loss of Siglec−14 reduces the risk of chronic obstructive pulmonary disease exacerbation. Cell Mol Life Sci. (2013) 70:3199–210. 10.1007/s00018-013-1311-7 23519826 PMC3718857

[103] Sewald X Ladinsky MS Uchil PD Beloor J Pi R Herrmann C. Retroviruses use CD169-mediated trans-infection of permissive lymphocytes to establish infection. Science (2015) 350:563–7. 10.1126/science.aab2749 26429886 PMC4651917

[104] Rempel H Calosing C Sun B Pulliam L. Sialoadhesin expressed on IFN-induced monocytes binds HIV-1 and enhances infectivity. PLoS ONE (2008) 3:e1967. 10.1371/journal.pone.0001967 18414664 PMC2288672

[105] Zou Z Chastain A Moir S Ford J Trandem K Martinelli E. Siglecs facilitate HIV-1 infection of macrophages through adhesion with viral sialic acids. PLoS ONE (2011) 6:e24559. 10.1371/journal.pone.0024559 21931755 PMC3169630

[106] Izquierdo-Useros N Lorizate M Puertas M Rodriguez-Plata M Zangger N Erikson E. Siglec-1 is a novel dendritic cell receptor that mediates HIV-1 trans-infection through recognition of viral membrane gangliosides. PLoS Biol. (2012) 10:e1001448. 10.1371/journal.pbio.1001448 23271952 PMC3525531

[107] Puryear W Akiyama H Geer S Ramirez N Yu X Reinhard B. Interferon-inducible mechanism of dendritic cell-mediated HIV-1 dissemination is dependent on Siglec-1/CD169. PLoS Pathog. (2013) 9:e1003291. 10.1371/journal.ppat.1003291 23593001 PMC3623718

[108] Varchetta S Lusso P Hudspeth K Mikulak J Mele D Paolucci S. Sialic acid-binding Ig-like lectin-7 interacts with HIV-1 gp120 and facilitates infection of CD4pos T cells and macrophages. Retrovirology (2013) 10:154. 10.1186/1742-4690-10-154 24330394 PMC3878752

[109] Suenaga T Satoh T Somboonthum P Kawaguchi Y Mori Y Arase H. Myelin-associated glycoprotein mediates membrane fusion and entry of neurotropic herpesviruses. Proc Natl Acad Sci USA. (2010) 107:866–71. 10.1073/pnas.0913351107 20080767 PMC2818916

[110] Suenaga T Matsumoto M Arisawa F Kohyama M Hirayasu K Mori Y. Sialic acids on varicella-zoster virus glycoprotein b are required for cell-cell fusion. J Biol Chem. (2015) 290:19833–43. 10.1074/jbc.M114.635508 26105052 PMC4528143

[111] Varchetta S Brunetta E Roberto A Mikulak J Hudspeth K Mondelli M. Engagement of siglec-7 receptor induces a pro-inflammatory response selectively in monocytes. PLoS ONE (2012) 7:e45821. 10.1371/journal.pone.0045821 23029261 PMC3461047

[112] Roy S Mandal C. Leishmania donovani utilize sialic acids for binding and phagocytosis in the macrophages through selective utilization of siglecs and impair the innate immune arm. PLoS Negl Trop Dis. (2016) 10:e0004904. 10.1371/journal.pntd.0004904 27494323 PMC4975436

[113] Yamanaka M Kato Y Angata T Narimatsu H. Deletion polymorphism of SIGLEC14 and its functional implications. Glycobiology (2009) 19:841–6. 10.1093/glycob/cwp052 19369701

[114] Bukvic BK Blekic M Simpson A Marinho S Curtin JA Hankinson J. Asthma severity, polymorphisms in 20p13 and their interaction with tobacco smoke exposure. Pediatr Allergy Immunol. (2013) 24:10–8. 10.1111/pai.12019 23331525

[115] Jonsson S Sveinbjornsson G de Lapuente Portilla AL Swaminathan B Plomp R Dekkers G. Identification of sequence variants influencing immunoglobulin levels. Nat Genet. (2017) 49:1182–91. 10.1038/ng.3897 28628107

[116] Hitomi Y Tsuchiya N Hasegawa M Fujimoto M Takehara K Tokunaga K. Association of CD22 gene polymorphism with susceptibility to limited cutaneous systemic sclerosis. Tissue Antigens (2007) 69:242–9. 10.1111/j.1399-0039.2007.00801.x 17493148

[117] Ma H Qazi S Ozer Z Gaynon P Reaman G Uckun F. CD22 Exon 12 deletion is a characteristic genetic defect of therapy-refractory clones in paediatric acute lymphoblastic leukaemia. Br J Haematol. (2012) 156:89–98. 10.1111/j.1365-2141.2011.08901.x 22017452

[118] Uckun F Goodman P Ma H Dibirdik I Qazi S. CD22 EXON 12 deletion as a pathogenic mechanism of human B-precursor leukemia. Proc Natl Acad Sci USA. (2010) 107:16852–7. 10.1073/pnas.1007896107 20841423 PMC2947921

[119] Naj A Jun G Beecham G Wang L Vardarajan B Buros J. Common variants at MS4A4/MS4A6E, CD2AP, CD33 and EPHA1 are associated with late-onset Alzheimer's disease. Nat Genet. (2011) 43:436–41. 10.1038/ng.801 21460841 PMC3090745

[120] Hollingworth P Harold D Sims R Gerrish A Lambert J Carrasquillo M. Common variants at ABCA7, MS4A6A/MS4A4E, EPHA1, CD33 and CD2AP are associated with Alzheimer's disease. Nat Genet. (2011) 43:429–35. 10.1038/ng.803 21460840 PMC3084173

[121] Malik M Simpson J Parikh I Wilfred B Fardo D Nelson P. CD33 Alzheimer's risk-altering polymorphism, CD33 expression, and exon 2 splicing. J Neurosci. (2013) 33:13320–5. 10.1523/JNEUROSCI.1224-13.2013 23946390 PMC3742922

[122] Raj T Ryan K Replogle J Chibnik L Rosenkrantz L Tang A. CD33: increased inclusion of exon 2 implicates the Ig V-set domain in Alzheimer's disease susceptibility. Hum Mol Genet. (2014) 23:2729-36. 10.1093/hmg/ddt666 24381305 PMC3990171

[123] Lamba JK Pounds S Cao X Downing JR Campana D Ribeiro RC. Coding polymorphisms in CD33 and response to gemtuzumab ozogamicin in pediatric patients with AML: a pilot study. Leukemia (2009) 23:402–4. 10.1038/leu.2008.185 18615103 PMC2659556

[124] Mortland L Alonzo T Walter R Gerbing R Mitra A Pollard J. Clinical significance of CD33 nonsynonymous single-nucleotide polymorphisms in pediatric patients with acute myeloid leukemia treated with gemtuzumab-ozogamicin-containing chemotherapy. Clin Cancer Res. (2013) 19:1620–7. 10.1158/1078-0432.CCR-12-3115 23444229 PMC3602123

[125] Wan C Yang Y Feng G Gu N Liu H Zhu S. Polymorphisms of myelin-associated glycoprotein gene are associated with schizophrenia in the Chinese Han population. Neurosci Lett. (2005) 388:126–31. 10.1016/j.neulet.2005.06.051 16039057

[126] Yang Y Qin W Shugart Y He G Liu X Zhou J. Possible association of the MAG locus with schizophrenia in a Chinese Han cohort of family trios. Schizophr Res. (2005) 75:11–9. 10.1016/j.schres.2004.11.013 15820319

[127] Jitoku D Hattori E Iwayama Y Yamada K Toyota T Kikuchi M. Association study of Nogo-related genes with schizophrenia in a Japanese case-control sample. Am J Med Genet B Neuropsychiatr Genet. (2011) 156B:581–92. 10.1002/ajmg.b.31199 21563301

[128] Munz M Willenborg C Richter GM Jockel-Schneider Y Graetz C Staufenbiel I. A genome-wide association study identifies nucleotide variants at SIGLEC5 and DEFA1A3 as risk loci for periodontitis. Hum Mol Genet. (2017) 26:2577–88. 10.1093/hmg/ddx151 28449029

[129] Liu H Irwanto A Fu X Yu G Yu Y Sun Y. Discovery of six new susceptibility loci and analysis of pleiotropic effects in leprosy. Nat Genet. (2015) 47:267–71. 10.1038/ng.3212 25642632

[130] Sun C Molineros JE Looger LL Zhou XJ Kim K Okada Y. High-density genotyping of immune-related loci identifies new SLE risk variants in individuals with Asian ancestry. Nat Genet. (2016) 48:323–30. 10.1038/ng.3496 26808113 PMC4767573

[131] Gao P Shimizu K Grant A Rafaels N Zhou L Hudson S. Polymorphisms in the sialic acid-binding immunoglobulin-like lectin-8 (Siglec-8) gene are associated with susceptibility to asthma. Eur J Hum Genet. (2010) 18:713–9. 10.1038/ejhg.2009.239 20087405 PMC2987348

[132] Laubli H Pearce O Schwarz F Siddiqui S Deng L Stanczak M. Engagement of myelomonocytic Siglecs by tumor-associated ligands modulates the innate immune response to cancer. Proc Natl Acad Sci USA. (2014) 111:14211–6. 10.1073/pnas.1409580111 25225409 PMC4191788

[133] Ishii T Angata T Wan ES Cho MH Motegi T Gao C. Influence of SIGLEC9 polymorphisms on COPD phenotypes including exacerbation frequency. Respirology (2017) 22:684–90. 10.1111/resp.12952 27878892

[134] McKay JD Hung RJ Han Y Zong X Carreras-Torres R Christiani DC. Large-scale association analysis identifies new lung cancer susceptibility loci and heterogeneity in genetic susceptibility across histological subtypes. Nat Genet. (2017) 49:1126–32. 10.1038/ng.3892 28604730 PMC5510465

[135] McDonough C Gong Y Padmanabhan S Burkley B Langaee T Melander O. Pharmacogenomic association of nonsynonymous SNPs in SIGLEC12, A1BG, and the selectin region and cardiovascular outcomes. Hypertension (2013) 62:48–54. 10.1161/HYPERTENSIONAHA.111.00823 23690342 PMC3686553

[136] Friedman E Moran DS Ben-Avraham D Yanovich R Atzmon G. Novel candidate genes putatively involved in stress fracture predisposition detected by whole-exome sequencing. Genet Res (Camb). (2014) 96:e004. 10.1017/S001667231400007X 25023003 PMC7045058

[137] Ma Q Ozel AB Ramdas S McGee B Khoriaty R Siemieniak D. Genetic variants in PLG, LPA, and SIGLEC 14 as well as smoking contribute to plasma plasminogen levels. Blood (2014) 124:3155–64. 10.1182/blood-2014-03-560086 25208887 PMC4231423

[138] Graustein AD Horne DJ Fong JJ Schwarz F Mefford HC Peterson GJ. The SIGLEC14 null allele is associated with Mycobacterium tuberculosis- and BCG-induced clinical and immunologic outcomes. Tuberculosis (Edinb). (2017) 104:38–45. 10.1016/j.tube.2017.02.005 28454648 PMC7289319

[139] Suhre K Arnold M Bhagwat AM Cotton RJ Engelke R Raffler J Connecting genetic risk to disease end points through the human blood plasma proteome. Nat Commun. (2017) 8:14357 10.1038/ncomms14357 28240269 PMC5333359

[140] Sun BB Maranville JC Peters JE Stacey D Staley JR Blackshaw J. Genomic atlas of the human plasma proteome. Nature. (2018) 558:73–9. 10.1038/s41586-018-0175-2 29875488 PMC6697541

[141] Sasayama D Hattori K Ogawa S Yokota Y Matsumura R Teraishi T. Genome-wide quantitative trait loci mapping of the human cerebrospinal fluid proteome. Hum Mol Genet. (2017) 26:44–51. 10.1093/hmg/ddw366 28031287

[142] Astle WJ Elding H Jiang T Allen D Ruklisa D Mann AL. The Allelic Landscape of Human Blood Cell Trait Variation and Links to Common Complex Disease. Cell. (2016) 167:1415–29 e19. 10.1016/j.cell.2016.10.042 27863252 PMC5300907

[143] Angata T. Associations of genetic polymorphisms of Siglecs with human diseases. Glycobiology (2014) 24:785–93. 10.1093/glycob/cwu043 24841380

[144] Martinez-Picado J McLaren PJ Erkizia I Martin MP Benet S Rotger M Identification of Siglec-1 null individuals infected with HIV-1x. Nat Commun. (2016) 7:12412 10.1038/ncomms12412 27510803 PMC4987525

[145] Martinez-Picado J McLaren PJ Telenti A Izquierdo-Useros N. Retroviruses As Myeloid Cell Riders: What Natural Human Siglec-1 “Knockouts” Tell Us About Pathogenesis. Front Immunol. (2017) 8:1593. 10.3389/fimmu.2017.01593 29209326 PMC5702442

[146] Buchlis G Odorizzi P Soto PC Pearce OM Hui DJ Jordan MS. Enhanced T cell function in a mouse model of human glycosylation. J Immunol. (2013) 191:228–37. 10.4049/jimmunol.1202905 23709682 PMC3691298

[147] Okerblom JJ Schwarz F Olson J Fletes W Ali SR Martin PT. Loss of CMAH during human evolution primed the monocyte-macrophage lineage toward a more inflammatory and phagocytic state. J Immunol. (2017) 198:2366–73. 10.4049/jimmunol.1601471 28148732 PMC5340615

[148] Okerblom J Varki A. Biochemical, cellular, physiological, and pathological consequences of human loss of N-glycolylneuraminic acid. Chembiochem (2017) 18:1155–71. 10.1002/cbic.201700077 28423240

[149] Varki A Altheide TK. Comparing the human and chimpanzee genomes: searching for needles in a haystack. Genome Res. (2005) 15:1746–58. 10.1101/gr.3737405 16339373

[150] Tangvoranuntakul P Gagneux P Diaz S Bardor M Varki N Varki A. Human uptake and incorporation of an immunogenic nonhuman dietary sialic acid. Proc Natl Acad Sci USA. (2003) 100:12045–50. 10.1073/pnas.2131556100 14523234 PMC218710

[151] Malykh YN Schauer R Shaw L. N-Glycolylneuraminic acid in human tumours. Biochimie (2001) 83:623–34. 10.1016/S0300-9084(01)01303-7 11522391

[152] Byres E Paton AW Paton JC Lofling JC Smith DF Wilce MC. Incorporation of a non-human glycan mediates human susceptibility to a bacterial toxin. Nature (2008) 456:648–52. 10.1038/nature07428 18971931 PMC2723748

[153] Moon JM Aronoff DM Capra JA Abbot P Rokas A. Examination of signatures of recent positive selection on genes involved in human sialic acid biology. G3 (Bethesda) (2018) 8:1315–25. 10.1534/g3.118.200035 29467190 PMC5873920

[154] Lewis A Desa N Hansen E Knirel Y Gordon J Gagneux P. Innovations in host and microbial sialic acid biosynthesis revealed by phylogenomic prediction of nonulosonic acid structure. Proc Natl Acad Sci USA. (2009) 106:13552–7. 10.1073/pnas.0902431106 19666579 PMC2726416

